# Desert particulate matter from Afghanistan increases airway obstruction in human distal lungs exposed to type 2 cytokine IL-13

**DOI:** 10.3389/fmed.2023.1177665

**Published:** 2023-06-28

**Authors:** Diana Cervantes, Niccolette Schaunaman, Gregory P. Downey, Hong Wei Chu, Brian J. Day

**Affiliations:** Department of Medicine, National Jewish Health, Denver, CO, United States

**Keywords:** particulate matter, human lung, IL-13, airway hyperresponsiveness, oxidative stress

## Abstract

**Introduction:**

Deployment related asthma-like symptoms including distal airway obstruction have been described in U.S. military personnel who served in Iraq and Afghanistan. The mechanisms responsible for the development of distal airway obstruction in deployers exposed to desert particulate matter (PM) is not well understood. We sought to determine if respiratory exposure to PM from Afghanistan (PMa) increases human distal airway hyperresponsiveness (AHR) with or without exposures to IL-13, a type 2 cytokine. We further tested whether mitochondrial dysfunction, such as ATP signaling and oxidative stress, may contribute to PMa- mediated AHR.

**Methods:**

Precision-cut lung slices from donors without a history of lung disease, tobacco smoking, or vaping were pre-treated with IL-13 for 24 h. This was followed by exposure to PMa or PM from California (PMc, control for PMa) for up to 72 h. The role of hydrogen peroxide and ATP in AHR was assessed using the antioxidant enzyme catalase or an ATP receptor P2Y13 antagonist MRS2211. AHR in response to methacholine challenges as well as cytokine IL-8 production were measured.

**Results:**

PMa alone, but not PMc alone, trended to increase AHR. Importantly, the combination of PMa and IL-13 significantly amplified AHR compared to control or PMc+IL-13. PMa alone and in combination with IL-13 increased IL-8 as compared to the control. PMa increased H2O2 and ATP. MRS211 and catalase reduced AHR in PCLS exposed to both PMa and IL-13.

**Discussion:**

Our data suggests that PMa in a type 2 inflammation-high lung increased AHR in part through oxidative stress and ATP signaling.

## Introduction

Nearly three million U.S. military personnel have served in Afghanistan and Iraq since 2001, of whom 14% reported deployment related asthma-like symptoms (1, 2). Environmental hazards from areas of deployment, such as sandstorms, burn bits, and combat dust, are linked to respiratory complications post deployment (2). Common respiratory reported include exertional dyspnea, cough, chest tightness, and wheezing (3). PM from each of these areas has a unique composition since there is variable exposure to diesel byproducts, metals, and other toxins (4). Regardless, parts of their surface composition, such as LPS, can act as Toll-like receptor ligands, which initiate an inflammatory response (5). Since PM is often coated with metals, PM exposures may lead to production of reactive oxygen species (ROS) and subsequent mitochondrial dysfunction (5, 6). The mitochondria are important for many normal cellular functions and are responsible for biosynthetic reactions such as ATP synthesis via the electron transport chain, ion homeostasis, and particularly Ca^2+^ regulation (7, 8). Loss of this homeostasis, often initiated by upstream stimuli like inflammation, ultimately leads to mitochondrial damage and induces a stress response (9). Mitochondrial dysfunction originating from mitochondrial damage or stress is associated with disease pathobiology such as asthma and chronic obstructive pulmonary disease (COPD) (10). Mitochondrial function can be altered by mitochondrial damage and oxidative stress induced by an increase in ROS production that include superoxide and hydrogen peroxide. Inflammation and ROS production can alter normal mitochondrial function. This initiates the release of mitochondrial DNA (mtDNA) and ATP, which serve as damage associated molecular patterns (DAMPS) (10). These DAMPS are released into the cytosol or extracellularly and can activate intracellular signaling cascades (10). An abnormal increase in ATP production can lead to a Ca^2+^ flux, activation of the inflammasome, further mtDNA release, increased ROS, and ongoing mitochondrial dysfunction (6, 8, 10). PM exposure diminishes the epithelial cell integrity which can activate Toll-like receptor signaling and induce oxidative stress and other downstream effects such as the release of pro-inflammatory cytokines (6, 8, 9, 11, 12).

A key feature of allergic asthma is airway hyperresponsiveness, which is characterized as narrowing of the airway lumen (13). The type 2 cytokine IL-13 plays a direct role in the recruitment of eosinophils during allergic inflammation, as well as in epithelial remodeling (14, 15). Manson et al. (13) showed that IL-13 induces airway hyperresponsiveness, which may be associated with increased ROS production and therefore leading to ER stress and an increase in Ca^2+^ flux levels in airway smooth muscle (16).

In the current study, given the role of PM or IL-13 in airway inflammation and AHR, we hypothesized that exposure to PMa alone and particularly in combination with IL-13 worsens distal airway obstruction in part through ROS and ATP signaling. We utilized human precision-cut lung slices (PCLS) as a highly physiologically relevant model to test our hypothesis.

## Methods

### Processing of human lungs for PLCS, and measurement of AHR

The upper lobes of the right lung from healthy non-smoking donors with no history of lung disease were obtained from the International Institute for the Advancement of Medicine (Philadelphia, PA, United States) or the Donor Alliance of Colorado (Denver, CO, United States). All the donor lungs were selected based on the non-smoking status and no history of lung disease/infection. The causes of death of the donors are related to car accidents, head trauma/brain bleeds, stroke or complications with heart conditions. The detailed donor demographic information is given in Table 1. The Institutional Review Board (IRB) at National Jewish Health approved our studies as meeting requirements of exempt human subject research.

**Table 1:** Demographics of human lung donors *n* = 5.

Subject	Age	Gender	Smoking status
1	30	Male	Non-smoker
2	27	Female	Non-smoker
3	20	Male	Non-smoker
4	75	Male	Former light smoker, quit 50 years ago
5	55	Male	Non-smoker

Human lungs were inflated with 1.5% low melting agarose (RPI, Mt. Prospect, IL) and cooled on ice. The tissue was cored and sliced using a Precisionary vibrating microtome at 300 μm thickness. The PCLS were incubated at 37°C in 24-well culture plates filled with 500 μL of DMEM containing penicillin/streptomycin, amphotericin B and fluconazole media with the addition of 0.2 M glutathione. Twenty-four hours after slicing, PCLS were pretreated with IL-13 (25 ng/mL). We chose IL-13 at 25 ng/mL because optimization on human PCLS showed greatest eotaxin 2 levels after 72 h. Previous studies have used IL-13 at 25 ng/mL in PCLS and IL-13 was able to induce AHR even after 5 days in culture (17). After 24 h of IL-13 treatment, PCLS were treated with particulate matter from Afghanistan (PMa) (95 ng/well) or particulate matter from California (PMc) (95 ng/well) in the presence or absence of an ATP receptor antagonist MRS2211 (1 μM) or catalase (500 U/mL), and IL-13 treatment was refreshed. We chose 95 μg/well because optimization done previously (11) showed that human airway epithelial cells exposed to PMa at 50 μg/cm^2^ had significantly higher IL-8 production with the least amount of cell death. Briefly, 50 μg/cm^2^ × 1.9 cm^2^ (area of 24 well plate) = 95ug/well. Notably, the 50 μg/cm^2^ is well in line with previous PM toxicity studies and a more physiologically relevant dose for *in vitro* modeling of PM exposures of deployed soldiers (11). The PCLS supernatants were collected for ATP, H_2_O_2,_ and IL-8 measurement 72 h post treatment. To measure AHR, baseline pictures of the airways were taken followed by adding 1, 10, 100, and 1,000 μM of methacholine (Mch) 30 s apart. Images of small airways (diameter < 2 μm) were taken between doses. The area (relative pixel number) within the lumen was traced using the image J freehand tool for all images, and the percent airway constriction was calculated as (1 - post Mch area/baseline area) × 100. The number of airways measured for AHR was between 3 and 9 per condition for every lung subject.

### Characterization of PMa and PMc

For PMa, Topsoil from Bagram Air Force Base in Parwan Province of Afghanistan was collected in August 2009 and characterized by the United States Geological Survey in Lakewood, Colorado. Collection and analysis were described previously by our group (11). The bulk topsoil was processed using a dry powder generator (Wright Dust Feeder, CH Technologies, Westwood, New Jersey) to produce respirable size of PM. Samples were analyzed via mass spectroscopy and scanning electron microscope for mineral composition as well as particle size and shape. Samples were found to contain calcite, dolomite, clay, kaolinite, illite, chlorite, muscovite, talc, biotite, feldspar, quartz, oxides, zircon, titanite, synchysite and monazite. The size of the PMa included 89.46% particles less than 2.5 μm, 10.4% between 2.5 μm and 10 μm, and 0.17% were greater than 10 μm.

For PMc, the topsoil (sand) from the area of China Lake, California was collected and aerosolized at NAMRU Dayton at the Wright Patterson air force base. Particles were run through a particle sizer and the resulting particles had a mean diameter of 1.91 μm. The settled particles were collected from the pan and then sterilized using gamma irradiation. Chemical analysis showed that PMc had similar crustal abundance levels to PMa.

### Measurement of ATP

An ATP colorimetric/Fluorometric assay kit (Sigma-Aldrich, St. Louis, MO) was used to quantify ATP levels in supernatants of human PCLS. The Promega GloMax explorer was used to measure the fluorescence intensity at 587 nm for 4 intervals every 30 min.

### Measurement of H_2_O_2_

The Invitrogen Amplex red hydrogen peroxide/peroxidase assay kit was used to quantify H_2_O_2_ levels in human PCLS supernatants. Samples were incubated at room temperature for 30 min in the dark, and fluorescence was measured using the Biotek microplate reader for a detection at 590 nm.

### Enzyme-linked immunosorbent assay

IL-8 levels were measured in PCLS supernatants using a Human IL-8 DuoSet ELISA kit (R&D Systems, Minneapolis, MN, United States) according to the manufacturer’s instructions.

### Statistical analysis

Parametric data were analyzed using paired *t*-tests for two group comparisons or one-way ANOVA analysis with Holm-Sidak’s *post hoc* test for multiple comparisons. Non-parametric data were analyzed using the Mann–Whitney test for two group comparisons. A *p* value < 0.05 was considered statistically significant.

## Results

### PMa enhanced airway hyperresponsiveness in IL-13-exposed human precision-cut lung slices

**Figure 1:**
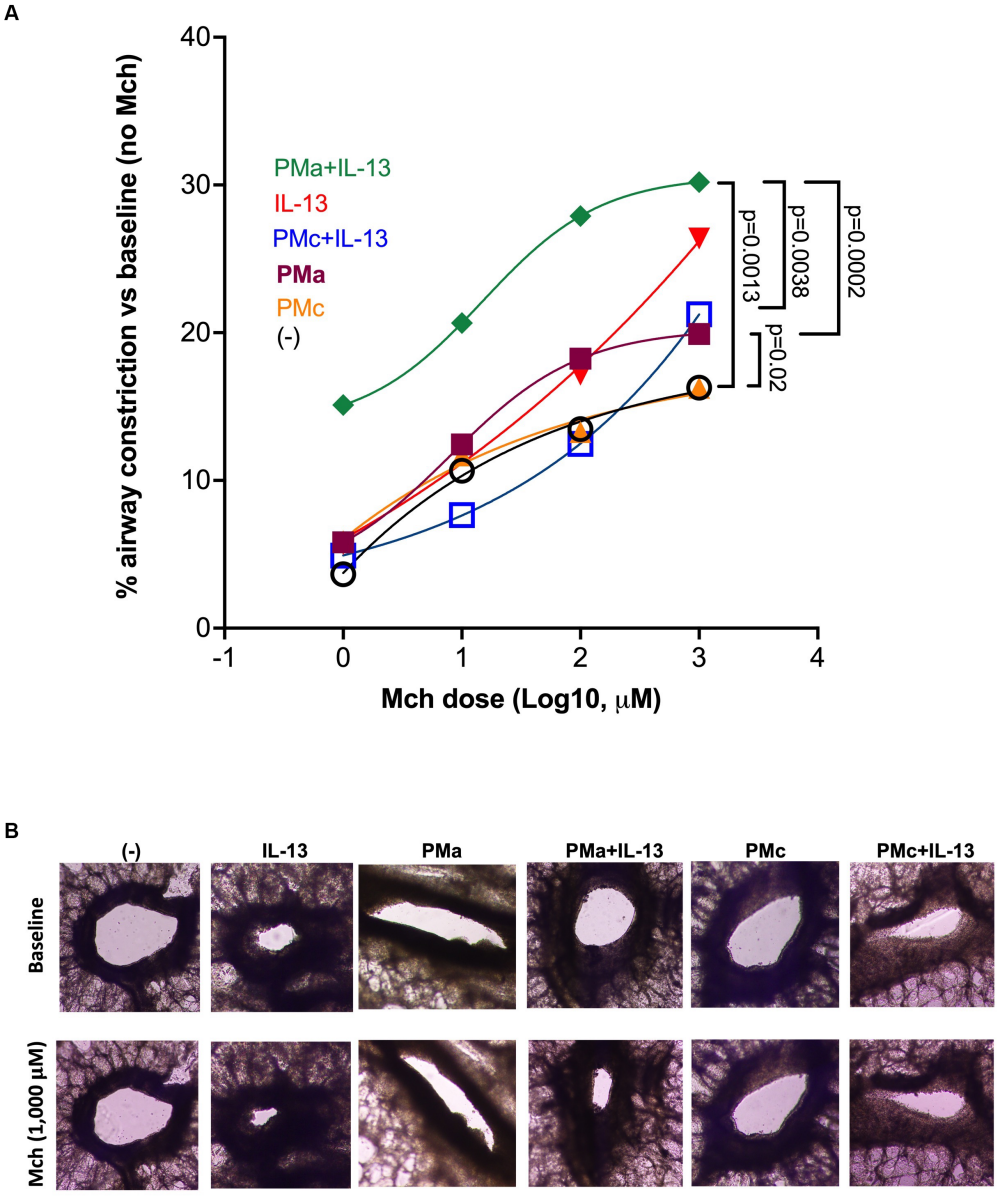
PMa increases airway constriction in human precision-cut lung slices exposed to IL-13. **(A)** After 72 h, PMa treated PCLS showed an increase in airway constriction and that was significantly increased with the addition of IL-13. Data is represented as a mean from 5 donors. PMa, PM from Afghanistan; PMc, PM from California. **(B)** Representative images from human precision-cut lung slices showing small airway constriction following methacholine (Mch) challenges under different treatment conditions. The images were taken under a phase contrast microscope at ×10 magnification.

Lung slices treated with PMa alone, as compared to the untreated ones, increased airway constriction in response to the methacholine challenge compared to the control. IL-13 alone trended to increase AHR but did not reach the level of statistical significance (*p* = 0.12). In the presence of both PMa and IL-13, AHR was further significantly increased (Figure 1). To test if PMa may have greater ability to induce AHR as compared to other PM, PMc was used as a control PM for PMa. Unlike PMa, PMc alone did not increase AHR. In the presence of IL-13, PMc was unable to significantly increase AHR (*p* = 0.26). Additionally, AHR in PMc-treated lung slices was significantly less than that in PMa-treated lung slices in the presence of IL-13 (Figure 1).

### Role of ATP signaling in AHR induced by PMa and IL-13

Extracellular ATP has been shown to contribute to airway inflammation and AHR in animal models of asthma (18, 19). In addition, previous studies have demonstrated that PM exposure results in mitochondrial dysfunction, such as mitochondrial damage, leading to less intracellular ATP, but more release of ATP into the extracellular space (20). Extracellular ATP binds to its receptors, such as P2Y13, to induce various biological effects (21).

**Figure 2:**
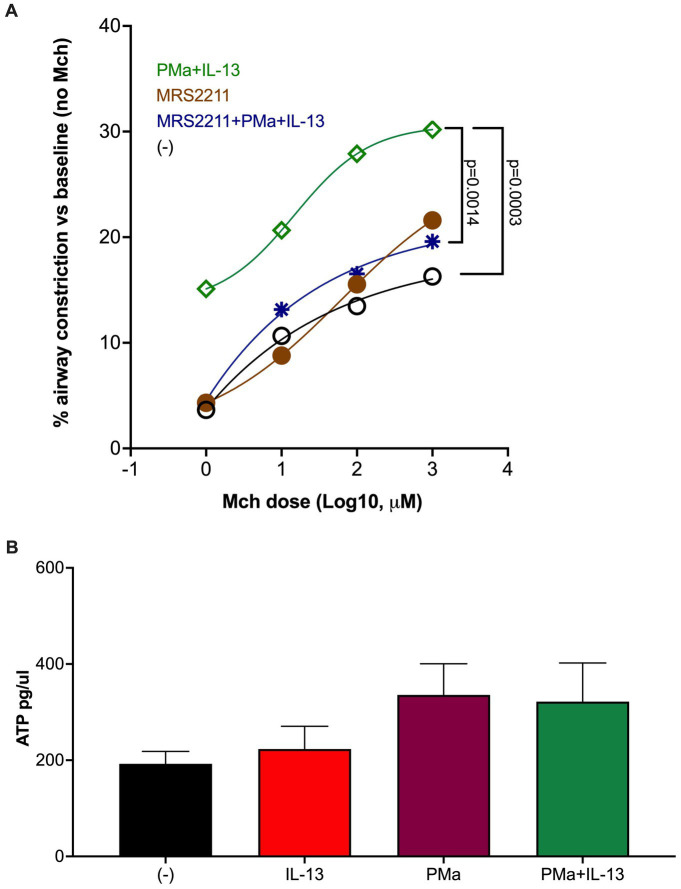
Blockade of ATP receptor P2Y13 signaling in human precision-cut lung slices reduces airway constriction. **(A)** MRS2211, a highly selective P2Y13 antagonist, significantly reduced AHR in the PCLS exposed to both PMa and IL-13. Data is presented as a mean from *n* = 5 donors. **(B)** ATP levels in supernatants of human PCLS. Data collected from 4 donors.

To determine if increased AHR by exposure to PMa and IL-13 may depends on ATP signaling, we used MRS2211, a highly selective antagonist of P2Y13 receptor, in lung slices. As shown in Figure 2A, MRS2211 significantly reduced AHR in slices treated with both PMa and IL-13. MRS2211 also reduced AHR in PMa-treated lung slices (data not shown).

Extracellular ATP was measured to examine the effect of PM and/or IL-13 on ATP release after 72 h of treatment. PMa alone and in combination with IL-13 trended to increase ATP (Figure 2B).

### Role of reactive oxygen species in AHR induced by PMa and IL-13 in human-precision cut lung slices

**Figure 3:**
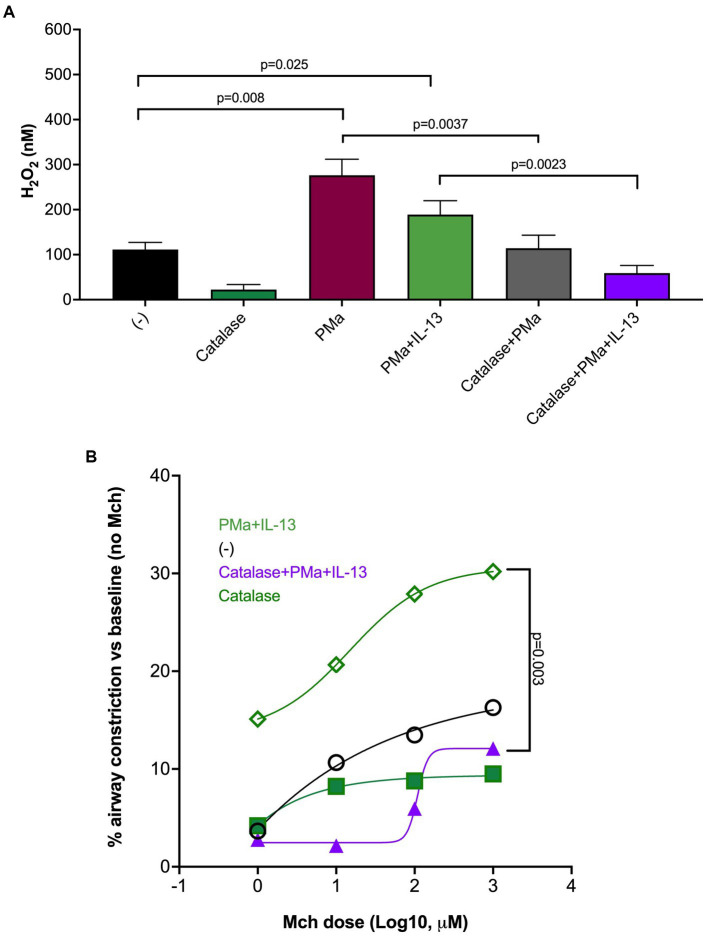
Catalase reduces airway constriction in human precision-cut lung slices exposed to IL-13 and PMa for 72 h. **(A)** PMa significantly increased H_2_O_2_ levels that were reduced by catalase. **(B)** Catalase significantly reduced airway constriction in the combination group (PMa+IL-13). Data is represented as mean from 3 donors (*n* = 10 replicates).

Oxidative stress, due to increased production of reactive oxygen species (e.g., H_2_O_2_), has been shown to contribute to mitochondrial dysfunction and ATP release induced by other sources of PM (22, 23). We measured H_2_O_2_ levels in supernatants of lung slices treated with PMa for 72 h in the absence or presence of catalase, an enzyme that converts H_2_O_2_ into water and oxygen. As shown in Figure 3A, H_2_O_2_ levels were higher following PMa treatments as compared to non-treated slices, which were not further increased by addition of IL-13. Nonetheless, H_2_O_2_ levels in lung slices treated with both PMa and IL-13 were still significantly higher than those in untreated lung slices (*p* = 0.025) (Figure 3). Catalase effectively in blocked H_2_O_2_ production induced by PMa alone or in combination of both PMa and IL-13. Catalase also reduced AHR induced by PMa in combination with IL-13 (Figure 3B).

### PMa and IL-13 increased IL-8 production in human-precision cut lung slices

**Figure 4:**
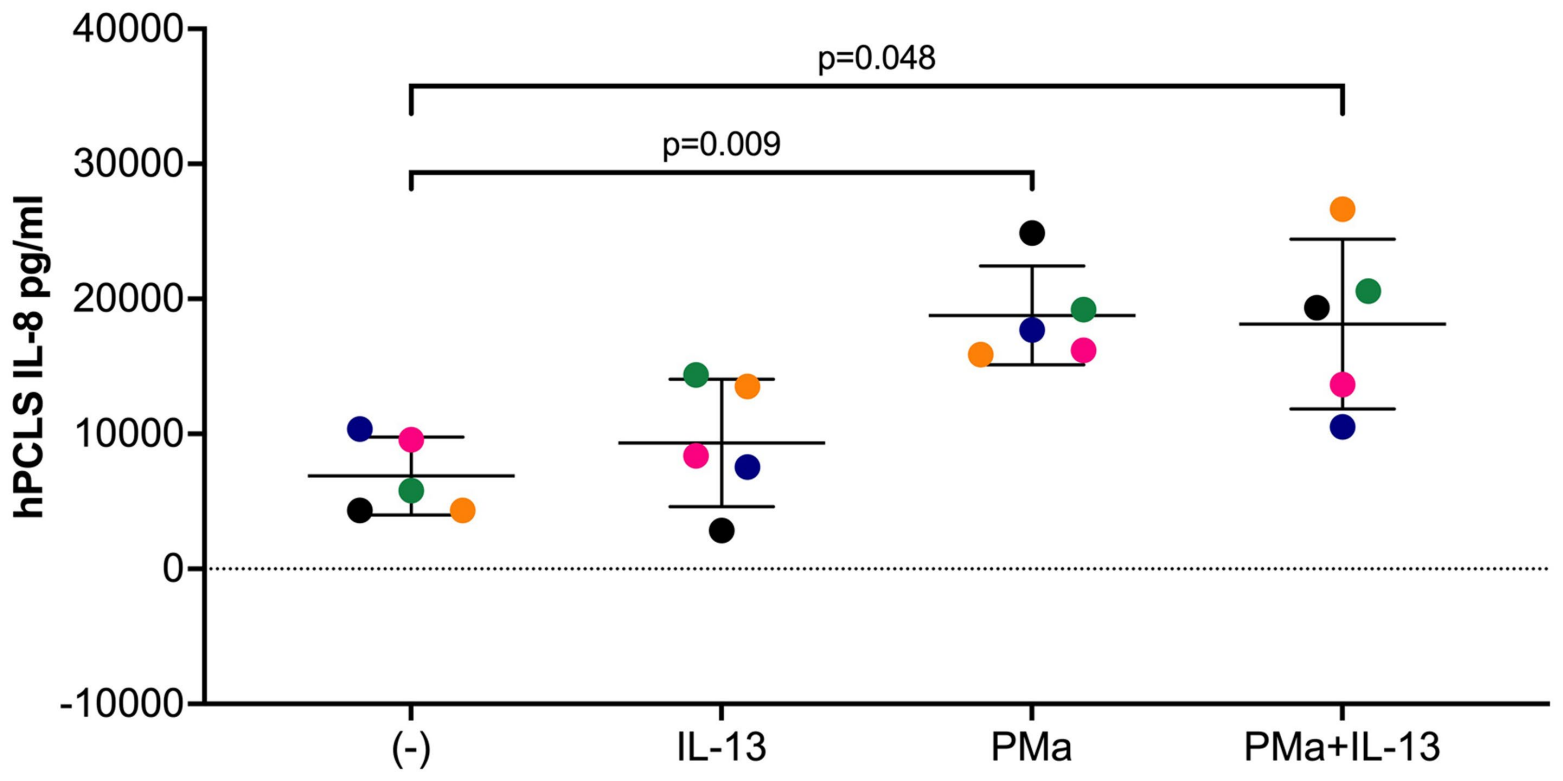
IL-8 levels in supernatants of human precision-cut lung slices exposed to IL-13 and/or PMa for 72 h. Data is represented as mean ± SEM from 5 donors. PMa, PM from Afghanistan; PMc, PM from California.

IL-8 is a chemokine for neutrophils that are associated with neutrophilic inflammation in lungs exposed to PM (24, 25). Interestingly, in cultured airway smooth muscle cells, IL-8 was shown to directly induce smooth muscle cell contraction (26), a key contributor to AHR. Here, we found that PMa alone, but not IL-13 alone, significantly increased the levels of IL-8 protein in the supernatants of lung slices (Figure 4). Although the combination of both PMa and IL-13 did not further increase IL-8 production, the levels of IL-8 in the combination group was still higher than those in the control group.

## Discussion

This study for the first time demonstrates that PMa exaggerated airway constriction in human distal lungs with high type 2 inflammation. Mechanistically, oxidative stress and mitochondrial dysfunction, such as ATP release and signaling, may contribute to worsened airway obstruction induced by both PMa and IL-13 exposures. Our improved understanding of PM-mediated airway obstruction may provide new insights into mechanisms of deployment-related respiratory disease and potential therapeutic approaches.

Various cell culture and animal models have been used to study the effect of PM on lung function. Previous studies in animals have shown that PM increases AHR in the absence or presence of allergen challenges (27, 28). However, there is a lack of direct evidence indicating that PM and in particular PMa, have an effect on airway obstruction in human lungs. Distal airways are the major site of pathology in deployment-related respiratory disease (3, 29). However, cell culture and mouse models do not fully represent the microenvironment in human distal lungs or cannot reflect the complexity of human physiological responses. The human lung PCLS model offers several major advantages over other models including intact tissue/organ architecture and native microenvironments (30–33). By using this unique PCLS model, we found that PMa alone significantly increased AHR that is consistent with previous animal studies on the health effects of PM (27, 34). Notably, when PMa was combined with IL-13, PMa further increased AHR. Our findings suggest an exacerbating effect of PMa on airway obstruction in the lungs with a type 2 cytokine-high background. Interestingly, we did not observe the exacerbating effect on AHR of PMc. PM can vary greatly in composition depending on many factors. PMa has a unique composition since it can be coated in burn pit combustion products and diesel exhaust particles, amongst other metals from explosive devises (5, 35). On the other hand, common PM’s composition is often associated with traffic-related air pollution (6). Whether the differences in composition of PMa and PMc contribute to the difference AHR response warrants future studies.

How PMa or PMa in combination with IL-13 increases AHR remains unclear. In the current study, we investigated two related mechanisms. First, we focused on ATP signaling as one of the potential mechanisms associated with mitochondrial dysfunction. We found that PMa increased extracellular ATP. While we saw an increase in extracellular ATP, this increase was not significant, which may be due to the short life of ATP being degraded at the time we measured the AHR. ATP release during tissue injury and inflammation is critical to the pathophysiology in asthma including AHR, neutrophilic inflammation, and Th17 response (19, 36). The blockade of ATP-releasing channels by ^10^Panx peptide has been shown to prevent increased extracellular ATP levels and AHR in an OVA-induced allergic asthma mouse model (37). We found that inhibition of ATP by a P2Y3 receptor antagonist MRS2211 reduced AHR in human PCLS exposed to both PMa and IL-13. Our data reveals that AHR induced by PMa and IL-13 may in part be dependent on ATP signaling. Second, we examined the role of ROS in AHR. ROS generation may be responsible for PM-mediated mitochondrial dysfunction (23). Frossi et al. (22) found that oxidative stress can upregulate type 2 inflammation contributing to AHR. We found that PMa increased H_2_O_2_ release. By using catalase to reduce H_2_O_2_ levels in human PCLS, we observed inhibition of oxidative stress reduced H_2_O_2_ levels and AHR in lung slices exposed to PMa and IL-13. Together our data suggest that mitochondrial dysfunction may be one of the mechanisms responsible for increased AHR by PMa and IL-13.

One limitation of this study is that we did not measure mitochondrial activity after PM exposure. Additionally, although we measured airway constriction in PM-exposed PCLS, we did not measure protein markers that may contribute to AHR. These proteins will be considered in our future experiments to improve our understanding of mechanisms by which PMa and IL-13 amplify AHR. We realize the sample size limitation in our study as there were 5 donors included. Nonetheless, all the donor demonstrated similar AHR responses following PMa and IL-13 treatment. Future studies using a larger group of healthy donors are warranted to extend our current study.

Overall, our findings offer novel mechanisms of distal airway obstruction in veterans that were previously deployed in Southeast Asia, and potential new therapies such as the use of antioxidants and ATP receptor antagonists to reduce the disease severity in deployers.

## Data availability statement

The original contributions presented in the study are included in the article/supplementary material, further inquiries can be directed to the corresponding authors.

## Ethics statement

The studies involving human participants were reviewed and approved by The Institutional Review Board (IRB) at National Jewish Health. Written informed consent for participation was not required for this study in accordance with the national legislation and the institutional requirements.

## Author contributions

DC, BD, and HC: conceptualization and investigation. DC, NS, and BD: methodology. DC and NS: validation. DC, NS, HC, and BD: formal analysis. HC, BD, and GD: resources and funding acquisition. DC and HC: data curation and writing—original draft preparation. DC, NS, GD, BD, and HC: writing—review and editing. DC, BD, GD, and HC: visualization. NS and HC: supervision. BD and HC: project administration. All authors contributed to the article and approved the submitted version.

## Funding

This work was funded by the Department of Defense (W81WH-16-2-0018).

## Conflict of interest

The authors declare that the research was conducted in the absence of any commercial or financial relationships that could be construed as a potential conflict of interest.

## Publisher’s note

All claims expressed in this article are solely those of the authors and do not necessarily represent those of their affiliated organizations, or those of the publisher, the editors and the reviewers. Any product that may be evaluated in this article, or claim that may be made by its manufacturer, is not guaranteed or endorsed by the publisher.
